# Ischemic stroke in a young patient with Fahr’s disease: a case report

**DOI:** 10.1186/s12883-016-0557-8

**Published:** 2016-03-08

**Authors:** Chih-Sheng Yang, Chung-Ping Lo, Man-Chun Wu

**Affiliations:** Department of Neurology, Taichung Tzu Chi Hospital, Buddhist Tzu Chi Medical Foundation, No 88, Sec 1, Fongsing Rd, Tanzi District Taichung City, 427 Taiwan; Department of Radiology, Taichung Tzu Chi Hospital, Buddhist Tzu Chi Medical Foundation, No 88, Sec 1, Fongsing Rd, Tanzi District Taichung City, 427 Taiwan; Department of Family Medicine, Taichung Tzu Chi Hospital, Buddhist Tzu Chi Medical Foundation, No 88, Sec 1, Fongsing Rd, Tanzi District Taichung City, 427 Taiwan

**Keywords:** Fahr’s disease, Basal ganglia calcification, Young stroke, Vascular calcification

## Abstract

**Background:**

Fahr’s disease is a rare neurodegenerative disorder characterized by diffuse intracranial calcium deposition and associated cell loss mainly in bilateral basal ganglia and dentate nuclei of the cerebellum. Subarachnoid hemorrhage and epileptic syncope had been reported as acute presentation of Fahr’s disease. We here report a 36-year-old male Indonesian diagnosed as Fahr’s disease presenting with young-onset ischemic stroke.

**Case presentation:**

A 36-year-old male Indonesian without prior systemic disease or neurologic disorder presented with young-onset ischemic stroke involving the right posterior limb of internal capsule. Brain computed tomography and magnetic resonance imaging demonstrated symmetric calcifications in bilateral basal ganglia, internal capsules, cerebellar dentate nuclei, thalami, cerebral white matter, which were all consistent with Fahr’s disease. The laboratory studies excluded the presence of other pathologic processes leading to secondary intracranial calcification. Other young stroke surveys were unremarkable. After medical treatment and sustained physical rehabilitation for 3 months, he recovered to carry out daily activities independently.

**Conclusion:**

We present ischemic stroke in a young patient with sporadic Fahr’s disease. The differentiation between Fahr’s disease and Fahr’s syndrome is specially highlighted when brain CT exhibits diffuse, symmetric calcifications in bilateral basal ganglia, thalami, cerebellar dentate nuclei and cerebral white matter. The association between nonarteriosclerotic vascular calcification and cerebrovascular disease is worth special attention and further investigation.

## Background

Fahr’s disease or idiopathic basal ganglia calcification (IBGC) is a rare neurodegenerative disorder characterized by diffuse, symmetric intracranial calcium deposition and associated cell loss mainly in bilateral basal ganglia and dentate nuclei of the cerebellum in the absence of other causes leading to secondary calcification. The disorder was first described by Karl Theodor Fahr in 1930 [1]. Patients usually present with chronic, progressive cognitive deterioration, psychiatric problems and extrapyramidal symptoms. The precise mechanism is not fully clear. It is postulated that calcifications in Fahr’s disease may be attributed to a metastatic deposition, secondary to local disruption of blood–brain barrier (BBB), or disorder of neuronal calcium phosphorus metabolism [2]. Fahr’s disease can manifest as autosomal dominant, familial or sporadic forms. Recent genetic research identified mutations in SLC20A2, a gene located in the IBGC3 region that encodes for type III sodium-dependent phosphate transporter 2 (PiT2) as a major cause for dominantly inherited Fahr’s disease [3]. In recent literatures, subarachnoid hemorrhage and epileptic syncope had been reported as acute presentation of Fahr’s disease [4, 5]. We here report a 36-year-old male Indonesian diagnosed as Fahr’s disease presenting with young-onset ischemic stroke. We review the literature and discuss the association between Fahr’s disease and cerebrovascular disease in this patient.

## Case presentation

**Fig. 1:**
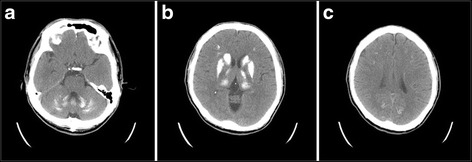
Axial noncontrast CT of the brain. Extensive and symmetric calcifications can be observed in **a** bilateral cerebellar dentate nuclei and hemispheres, **b** bilateral basal ganglia, internal capsules, and thalami, and **c** bilateral cerebral subcortical white matter

A 36-year-old male Indonesian who denied prior systemic disease or neurologic disorder had been healthy and came to Taiwan for 8 months, working as a labor of electronic factory. No family history of cognitive impairment, movement disorder or young-onset ischemic stroke was obtained. He presented to our emergency department because of acute onset of left limb weakness when he was working on Nov 18th, 2014. At emergency room, the initial vital signs were: blood pressure 156/95 mmHg, pulse 91 beats/min, respirations 16 breaths/min, body temperature 36 °C. The general physical examination was unremarkable. No cardiac arrhythmia, heart murmur or carotid bruit was observed. The neurologic examination revealed left hemiparesis with manual muscle power grade 4, mild dysarthria and left central facial palsy. There was no cognitive impairment, extrapyramidal signs or cerebellar ataxia. The score of National Institutes of Health Stroke Scale (NIHSS) was four points. Brain computed tomography (CT) showed extensive and symmetric calcifications involving the bilateral basal ganglia, internal capsules, thalami, cerebral subcortical white matter, cerebellar dentate nuclei and deep cerebellar white matter (Fig. 1). The blood examinations at emergency room were normal including coagulation profiles, blood cell count and biochemistry profiles. He was admitted to neurologic ward with the tentative diagnosis of young-onset ischemic stroke.

**Fig. 2:**
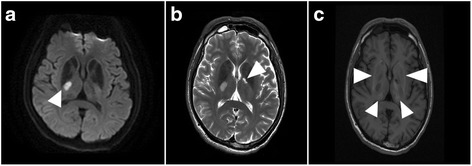
The findings of brain MRI. **a** Acute ischemic infarction involving the right posterior limb of internal capsule presented as hyperintense area in diffusion weighted images (*arrowhead*). **b** An old lacunar infarction involving the left genu of the internal capsule was seen in T2 weighted images (*arrowhead*). **c** Nonenhanced T1 weighted images showed hyperintense signal in bilateral basal ganglia, thalami (*arrowheads*) and cerebellar dentate nuclei (not shown)

**Fig. 3:**
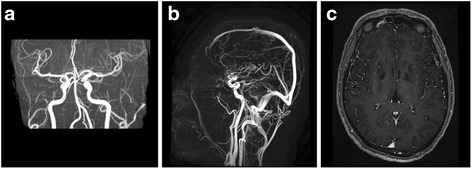
**a** Magnetic resonance angiography and **b** venography. No intracranial large-vessel stenosis, aneurysm, vascular malformation or dural sinus thrombosis was observed. **c** The contrast-enhanced T1 weighted images showed no abnormal parenchymal or leptomeningeal enhancement

**Fig. 4:**
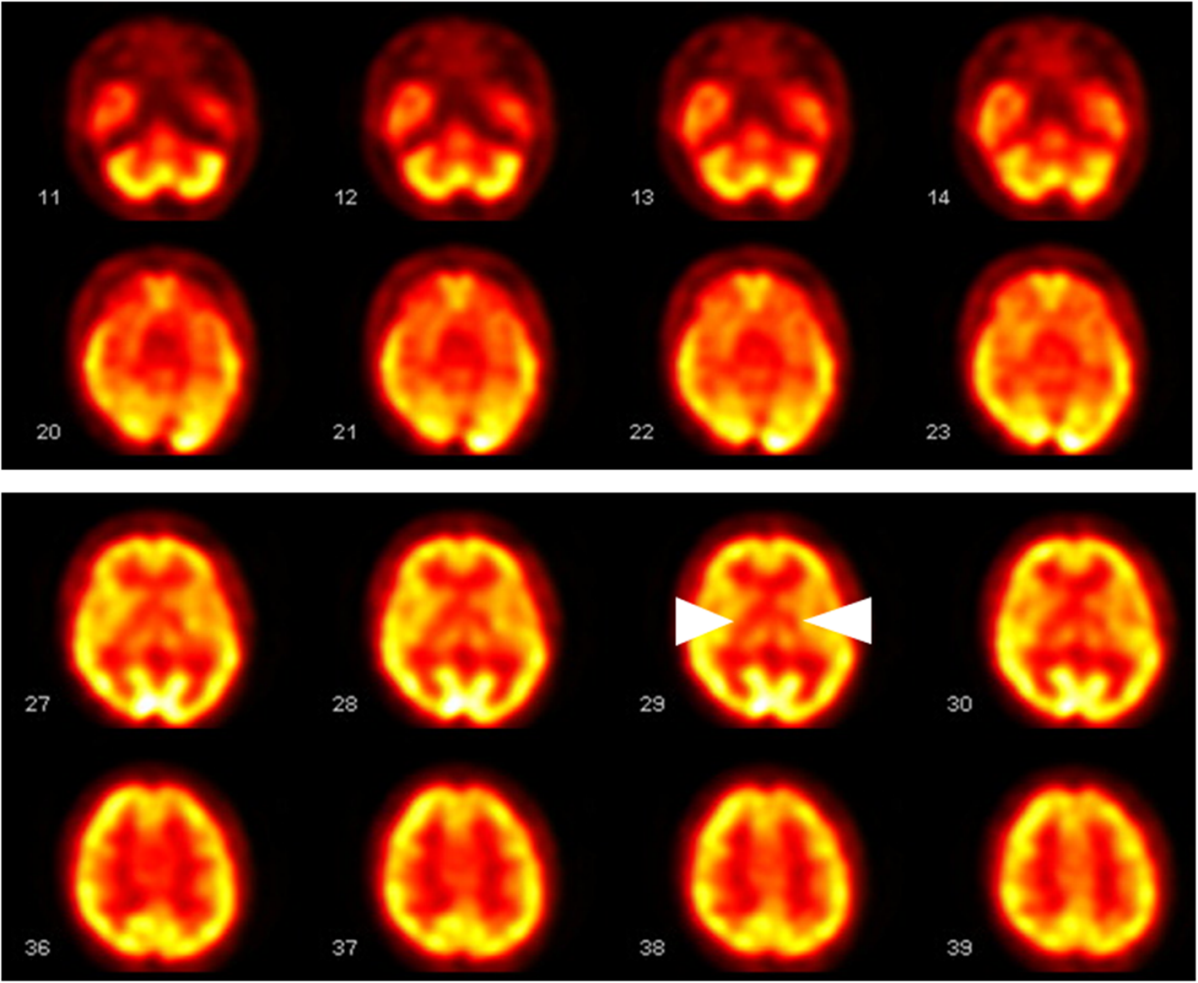
The features of cerebral perfusion SPECT. The presence of hypoperfusion in the bilateral basal ganglia and thalamic regions (*arrowheads*) as well as heterogeneous radiouptake in the bilateral cerebral hemispheres and cerebellar hemispheres corresponded to those calcified areas in brain CT

At hospitalization, the causes of abnormal intracranial calcification and young-onset stroke were investigated. The level of serum calcium (2.20 mmol/L), phosphorus (4.3 mg/dL), magnesium (2.1 mg/dL), alkaline phosphatase (79 IU/L), calcitonin, thyroid hormone, parathyroid hormone and enzyme-linked immunosorbant assay for human immunodeficiency virus (HIV) were all within normal limits. Brain magnetic resonance imaging (MRI) revealed acute ischemic infarction involving the right posterior limb of internal capsule and an old lacunar infarct involving the left genu of the internal capsule. In addition, hypointense signal in T2 weighted images and slightly hyperintense signal in T1 weighted images were identified at bilateral basal ganglia, thalami, and cerebellar dentate nuclei corresponding to the calcified lesions (Fig. 2). Magnetic resonance angiography and venography excluded intracranial large-vessel stenosis, aneurysm, vascular malformation or dural sinus thrombosis. The contrast-enhanced MRI excluded abnormal parenchymal or leptomeningeal enhancement (Fig. 3). Cerebral perfusion single-photon emission computed tomography (SPECT) of the brain with 99mTc-hexamethylpropyleneamine oxime (99mTc-HMPAO) demonstrated the presence of hypoperfusion in the bilateral basal ganglia and thalamic regions as well as heterogeneous radiouptake in the bilateral cerebral hemispheres and cerebellar hemispheres (Fig. 4). The young stroke surveys including carotid artery duplex, transthoracic cardiac echo, 24-h Holter, hematologic measurements, D-dimer level (213.23 ng/mL), coagulation profiles, autoimmune profiles, inflammation indices were all unremarkable.

Aspirin with doses of 200 mg daily and adequate hydration was regularly administered. However, his left limb weakness started to deteriorate on day 2 after admission with NIHSS score rising to six points. There was no other cortical function involvement. For stroke in evolution, low-molecular-weight heparin with Dalteparin 2500 IU subcutaneously every 12 h had been given for 5 days and his left hemiplegia gradually stabilized. After sustained physical rehabilitation for 3 months, he recovered to carry out daily activities independently. The score of NIHSS at discharge was two points. The treatment resulted in favorable functional outcome that was assessed by the modified Rankin Scale score of 1 and the Barthel Index score of 100.

## Conclusion

Fahr’s disease or idiopathic basal ganglia calcification is a rare neurologic disorder with variable clinical presentations and distinctive neuroradiological features. According to a registry of Fahr’s disease, symptomatic individuals accounted for 67 % of the total population. Of the symptomatic cases, the incidence among men was higher compared with women and movement disorder was the most common manifestation [6]. Brain CT remains a sensitive tool to detect this excessive intracranial calcification because the calcium deposits are distinctly hyperdense. However, the signal intensity is variable on MRI and depends on the proton density of calcium and other mineral ions, the concentration of binding proteins and mucopolysaccharides in different stages of the disease or different metabolic states [7]. The common sites of calcifications in Fahr’s disease are globus pallidus, putamen, caudate nucleus, internal capsule, dentate nucleus, thalamus, and cerebral white matter [8]. In our patient, brain MRI demonstrated different patterns in these calcified regions. In T1 weighted images, hyperintense signals were only recognized in bilateral basal ganglia, thalami and cerebellar dentate nuclei.

Fahr’s disease needs to be distinguished from Fahr’s syndrome in which basal ganglia calcification is attributed to secondary cause. The differential diagnosis of pathologic basal ganglia calcification include idiopathic hypoparathyroidism, secondary hypoparathyroidism, hyperparathyroidism, post-thyroidectomy, birth anoxia, cysticercosis, toxoplasmosis, calcified infarct, HIV infection…etc. [9]. In general, the distribution of intracranial calcification tends to be extensive and symmetric in endocrine, toxic, metabolic or degenerative etiologies. The pathologic calcified lesions secondary to infectious disease, vascular insults or neoplasm are usually scattered and asymmetric in the size and location [10, 11]. Detailed history taking and laboratory investigations are helpful in determining the etiologies of pathologic intracranial calcification. As for our patient, he had been well until this event and does not have developmental anomaly, infection signs or toxin exposure. He had no systemic disease, metabolic disorder or hypoxia history. The thorough laboratory studies excluded the presence of other pathologic processes. Brain CT, supplemented by MRI study exhibited symmetric calcifications in the bilateral basal ganglia, thalami, bilateral cerebral subcortical white matter, cerebellar dentate nuclei and deep cerebellar white matter, which were all consistent with Fahr’s disease. The patient denied previous familial illness. Therefore, we considered him a sporadic case of Fahr’s disease.

The association between young-onset ischemic stroke and Fahr’s disease has yet to be determined. Transient ischemic attack-like episodes had been linked to Fahr’s disease in two case reports [12, 13]. However, there have been no case reports demonstrating acute infarction in Fahr’s disease with positive MRI findings and describing the association with young-onset ischemic stroke. Besides, this is the first case report of Fahr’s disease presenting with ischemic stroke in Asian population. In most cases with Fahr’s disease, although the age at onset of neuropsychiatric symptoms is fourth to sixth decades of life, highly variable clinical manifestations were also reported in childhood-onset case, indicating phenotypic heterogeneity and different functional impairment in young-aged population [14]. In addition, the clinical presentations are also correlated with the calcified sites, the amount of calcifications and consequently changes in functional circuits [6, 15].

Due to uncertainty regarding the exact pathogenesis, a plethora of descriptive terms have been used to describe this disease. Different names of Fahr’s disease such as idiopathic nonarteriosclerotic calcification of cerebral vessels and idiopathic familial cerebrovascular ferrocalcinosis reflect the etiology with the predilection of cerebral vascular involvement [16]. Previous pathologic studies showed calcium and other mineral deposits found in the walls of capillaries, arterioles, small veins and in the perivascular spaces [17]. Electron microscopic study in post mortem cases also revealed calcium deposits in the cytoplasm of adventitial cells of blood vessels and sometimes in the cytoplasmic processes of glial cells [18]. In addition, neuronal degeneration and gliosis surrounding these mineral accumulations have been reported [19]. In many studies of coronary artery disease, vascular calcification compromises arterial elastance and vasomotor responses, leading to unstable angina and myocardial infarction. Thus, vascular calcification is regarded as an important risk factor of coronary heart disease [20]. In our case, a young healthy male had recurrent ischemic infarction involving the bilateral internal capsules respectively corresponding to the territory of smaller vessels such as anterior choroidal arteries or perforating arteries. These infarct lesions are deep and smaller than 1.5 cm in diameter. We made the diagnosis of pure motor lacunar syndrome and classified the etiologic subtype of ischemic stroke as small-artery occlusion based on the Trial of Org 10172 in Acute Stroke Treatment (TOAST) classification system [21]. Furthermore, the neuroimaging features that support cardioembolic stroke or malignancy-related embolic infarction, such as stroke in multiple vascular territories, combined anterior and posterior circulation or bilateral/multilevel posterior infarcts, were not observed in our patient’s pictures. Other ancillary diagnostic studies excluded high risk sources of cardioembolism and autoimmune disease-related ischemic stroke. Normal D-dimer level and lacunar infarction appearance make the possibility of malignancy-related embolic infarction less likely [22]. We therefore postulate that the underlying pathogenic process of Fahr’s disease results in extensive calcium and mineral deposits in affected vessels and subsequently predisposes these subjects to young-onset cerebrovascular disease. This presumptive pathogenic mechanism is quite distinct from other conventional etiologies that affect the small vessels including arteriosclerosis, cerebral amyloid angiopathy and immunologically-mediated small vessel diseases. Cerebral perfusion SPECT of the brain with 99mTc-HMPAO is useful in evaluating regional blood flow [23]. Reduced blood flow to calcified regions is documented in our patient, which may lead to further focal ischemic change.

There is no specific treatment for Fahr’s disease to limit the progression of brain calcification. Treatment is usually symptomatic. The trial of treatment with central nervous system-specific calcium channel blocker such as nimodipine failed to confirm benefit [6]. In some preliminary studies, disodium etidronate, a biphosphonate exhibited functional benefit and symptomatic improvement without reduction in the amount of calcifications [24, 25]. We treated the patient with antiplatelet agent initially and combined anticoagulant agent during the stage of stroke in evolution. The final neurologic outcome is good. Nevertheless, the true prevalence of acute ischemic stroke in Fahr’s disease remains unknown due to lack of large-scale study.

In summary, we present ischemic stroke in a young patient with sporadic Fahr’s disease. The differentiation between Fahr’s disease and Fahr’s syndrome is specially highlighted when brain CT shows diffuse, symmetric calcifications in bilateral basal ganglia, thalami, cerebellar dentate nuclei and cerebral white matter. The association between nonarteriosclerotic vascular calcification and cerebrovascular disease is worth special attention and further investigation.

### Consent

Written informed consent was obtained from the patient for publication of this case report and accompanying images. A copy of the written consent is available for review by the Editor-in-Chief of this journal.

## Abbreviations

99mTc-HMPAO: 99mTc-hexamethylpropyleneamine oxime.
BBB: blood–brain barrier
CT: computed tomography
HIV: human immunodeficiency virus
IBGC: idiopathic basal ganglia calcification
MRI: magnetic resonance imaging
NIHSS: National Institutes of Health Stroke Scale
SPECT: single-photon emission computed tomography
